# Isolation, identification, molecular typing, and drug resistance of *Escherichia coli* from infected cattle and sheep in Xinjiang, China

**DOI:** 10.1002/vms3.1101

**Published:** 2023-03-28

**Authors:** Xiaoxiao Gu, Xue Ma, Qin Wu, Qiaoxiaoci Tao, Yingjin Chai, Xia Zhou, Mengli Han, Jie Li, Xin Huang, Tongzhong Wu, Xingxing Zhang, Fagang Zhong, Yiheng Cao, Liyuan Zhang

**Affiliations:** ^1^ College of Animal Science and Technology Shihezi University Shihezi China; ^2^ State Key Laboratory for Sheep Genetic Improvement and Healthy Production Xinjiang Academy of Agricultural and Reclamation Science Shihezi China

**Keywords:** antibiotic resistance, cattle, *Escherichia coli* infections, multiplex polymerase chain reaction, phylogeny, virulence factors, sheep

## Abstract

**Background:**

*Escherichia coli* infections are common in Xinjiang, a major region of cattle and sheep breeding in China. Therefore, strategies are required to control *E. coli*. The aim of this study was to investigate the phylogenetic groups, virulence genes, and antibiotic resistance characteristics of *E. coli* isolates.

**Methods:**

In this study, 116 tissue samples were collected from the organs of cattle and sheep that were suspected of having *E. coli* infections between 2015 and 2019. Bacteria in the samples were identified using a biochemical identification system and amplification of 16S rRNA, and the phylogenetic groupings of *E. coli* isolates were determined by multiplex polymerase chain reactions. In addition, PCR detection and analysis of virulence factors, antibiotic resistance genes, and drug‐resistant phenotypes of *E. coli* isolates were performed.

**Results:**

A total of 116 pathogenic *E. coli* strains belonging to seven phylogenetic groups were isolated, with the majority of isolates in groups A and B1. Among the virulence genes, curli‐encoding *crl* had the highest detection rate of 97.4%, followed by hemolysin‐encoding *hlyE* with the detection rate of 94.82%. Antimicrobial susceptibility test results indicated that the isolates had the highest rates of resistance against streptomycin (81.9%).

**Conclusion:**

These characteristics complicate the prevention and treatment of *E. coli*‐related diseases in Xinjiang.

## Graphical abstract

The prevalence of multi‐antibiotic‐resistance, and virulence gene carrier rate were generally high among *E. coli* isolates from cattle and sheep suspected of *E. coli* disease in Xinjiang, and is increasing year by year. The virulence gene carrying rate of cattle strains is much higher than that of sheep strains.

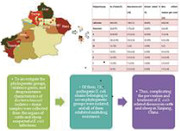

## 1 INTRODUCTION

*Escherichia coli* isolates can be classified based on their virulence factors as intestinal pathogenic *E. coli*, extraintestinal pathogenic *E. coli* (ExPEC), or symbiotic *E. coli* (Liu et al., 2017; Wirth et al., 2006). Pathogenic *E. coli*, a key zoonotic pathogen, is responsible for more than 160 million cases of dysentery and 1 million deaths every year (Guan & Li, 2007). Currently, *E. coli* strains are assigned to eight phylogenetic groups of isolates with different pathogenicities based on genetic and evolutionary characteristics: A, B1, B2, C, D, E, F, and clade I (Bok et al., 2020; Clermont et al., 2013; Ksiezarek et al., 2021). The pathogenicity of *E. coli* is determined by regulatory and interactive effects among various virulence factors and is affected by host type, host health, and interactions with other bacteria. Excrements of cattle and sheep commonly contain both pathogenic and non‐pathogenic *E. coli*, which enables the rapid transmission of *E. coli* across livestock populations (Chase‐Topping et al., 2012; Busia et al., 2014; Reiss et al., 2006; Sargeant et al., 2004). Other studies have shown that pathogenic *E. coli* isolates originating from the intestinal tracts of cattle and sheep may infect humans (Irino et al., 2007). Furthermore, selective pressure from the widespread use of antibiotics in livestock breeding has resulted in an increase in bacterial resistance against antibacterial agents, as well as cross‐resistance to other agents (Roth et al., 2019; Tasho et al., 2016). Multidrug antibiotic resistance in bacteria is a severe threat to human health (Bai et al., 2021). In 2019, antimicrobial resistance was named by the World Health Organization (WHO) as one of the top 10 threats to global health (WHO, 2019). Because *E. coli* infections are common in cattle and sheep breeding industries of Xinjiang, tissue samples (liver, spleen, lungs, heart and lymph nodes) were collected from 2015 to 2019 from infected cattle and sheep from breeding farms in Xinjiang for the isolation and identification of *E. coli* strains. The phylogenetic groups, virulence genes, and antibiotic resistance characteristics of these isolates were investigated to formulate strategies to prevent and control *E. coli* infections within cattle and sheep populations in the region.

## 2 METHODS

### 2.1 Sample collection and *E. coli* isolation and identification

Over 4 years (2015–2019), lung, spleen, heart, and lymphoid tissue samples were collected from cattle and sheep that manifested the following symptoms: diarrhoea, elevated body temperature, listlessness, limb weakness, neurological symptoms, sepsis, and evidence of respiratory tract infection. In the sampling process, we collected the fresh tissue using sterile surgical instruments, and then placed them in 10 mL aseptic tubes. The samples were placed on ice packs and later transported to the laboratory within 24 h of collection for bacterial isolation. The samples were inoculated into a nutrient broth and cultured at 37°C and 180 rpm for 6–8 h. Subsequently, the cultures were inoculated onto eosin‐methylene blue agar, MacConkey agar, and blood agar plates and cultured at 37°C for 10–15 h. In addition, genes were purified and sequenced using 16S rRNA, verifying the possibility of certain bacterial species (Clarridge, 2004). Suspected *E. coli* colonies on each agar plate were purified and identified using an automated microbial identification system (VITEK 2 Compact; bioMérieux, France) (Gu et al., 2018). All animal experiments were performed according to the Chinese Regulations of Laboratory Animals—The Guidelines for the Care of Laboratory Animals (Ministry of Science and Technology of People's Republic of China) and Laboratory Animal Requirements of Environment and Housing Facilities (GB 14925–2010, National Laboratory Animal Standardization Technical Committee). The animal experiments were reviewed and approved by the ethics committee of the Hospital (approval number: A2019‐149‐01).

### 2.2 DNA extraction

*Escherichia coli* cells were streaked onto Luria‐Bertani agar plates(Sangon, China), cultured at 37°C for 18 h, and inoculated into a nutrient broth for culture at 37°C and 180 rpm for 18 h (Böhnlein et al., 2016). Genomic DNA was extracted in accordance with bacterial DNA extraction kit instructions(Qiagen, Germany), and the extracted DNA was stored at −20°C for further use.

### 2.3 Detection of virulence determinants by polymerase chain reactions and phylogenetic group determination

The *E. coli* phylogroup (A, B1, B2, C, D, E, and F) of each isolate was determined by the polymerase chain reactions (PCR) quadruplex trpA and arpA allele‐specific amplification methods (Clermont et al., 2013) (Table 1).

**TABLE 1:** Primer sequences for quadruplex phylogrouping of *E. coli* isolates.

Gene	Primer name	Primer sequence (5′→3′)	Length (bp)	Reference
*16S rRNA*	16S rRNA F	AGAGTTTGATCMTGGCTCAG	1500	(Clermont et al., 2013)
	16S rRNA R	TACGGYTACCTTGTTACGACTT		
*arpA* (quadruplex)	arpAF arpA R	AACGCTATTCGCCAGCTTGC	400	(Clermont et al., 2013)
		TCTCCCCATACCGTACGCTA		
*chuA*	chuA F	GACGAACCAACGGTCAGGAT	288	(Clermont et al., 2013)
(quadruplex)	chuAR	TGCCGCCAGTACCAAAGACA		
*yjaA*	yjaA F	TGAAGTGTCAGGAGACGCTG	211	(Clermont et al., 2013)
(quadruplex)	yjaA R	ATGGAGAATGCGTTCCTCAAC		
*TspE4.C2*	TspE4.C2F	GAGTAATGTCGGGGCATTCA	152	(Clermont et al., 2013)
(quadruplex)	TspE4.C2 R	GCGCCAACAAAGTATTACG		
Group C *trpA*	GroupCtrpA F	AGTTTTATGCCCAGTGCGAG	219	(Clermont et al., 2013)
	GroupCtrpAR	TCTGCGCCGGTCACGCCC		
Group E *arpA*	GroupEarpA F	GATTCCATCTTGTCAAAATATGCC	301	(Clermont et al., 2013)
	GroupEarpA R	GAAAAGAAAAAGAATTCCCAAGAG		
Internal control *trpA*	Internal control F	CGGCGATAAAGACATCTTCAC	489	(Clermont et al., 2013)
	Internal control R	GCAACGCGGCCTGGCGGAAG		

PCR detection of virulence genes in *E. coli* originating from cattle and sheep, including *crl*, *agg*, *stx1*, *stx2*, *fimH*, *hlyA*, *hlyE*, *astA*, *ibeA*, *iucD*, *kpsMII*, *pap*, *cnfI*, *sfaS*, *malX*, *ompA*, *ompC*, *ompF*, *K88*, *K99*, and *eaeA* was performed using methods described by Antikainen et al. (2009), Kotlowski et al. (2007), Wang et al. (2012), and Yue et al. (2017). PCRs were carried out in a 25 μL reaction mixture consisting of 12.5 μL of 2× mix, 8.5 μL of ddH_2_O, 1 μL each of forward and reverse primers (10 μmol/mL), and 2 μL of DNA template. The PCR cycling conditions are shown in Table 2.

**TABLE 2:** Primers used to detect *E. coli* virulence genes.

Gene	Primer	Primer sequence (5′→3′)	Length (bp)	Annealing temperature (°C)	Reference
*crl*	crlF	TTTCGATTGTCTGGCTGTATG	250	53	(Wang, 2012)
	crlR	CTTCAGATTCAGCGTCGTC			
*agg*	aggF	ACGCAGAGTTGCCTGATAAAG	400	55	(Antikainen et al., 2009)
	aggR	ACGCAGAGTTGCCTGATAAAG			
*stx1*	stx1F	TTCGCTCTGCAATAGGTA	555	50	(Wang, 2012)
	stx1R	TTCCCCAGTTCAATGTAAGAT			
*stx2*	stx2F	GTGCCTGTTACTGGGTTTTTCTTC	118	56	(Wang, 2012)
	stx2R	GTGCCTGTTACTGGGTTTTTCTTC			
*fimH*	fimHF	CTGGTCATTCGCCTGTAAAACCGCCA	466	50	(Kotlowski et al., 2007)
	fimHR	GTCACGCCAATAATCGATTGCACATTCCCT			
*hlyA*	hlyAF	TGCAGCCTCCAGTGCATCCCTC	355	62	(Kotlowski et al., 2007)
	hlyAR	CTTACCACTCTGACTGCGATCAGC			
*hlyE*	hlyEF	GAAACCGCAGATGGAGCATT	553	56	(Wang, 2012)
	hlyER	CGCCCGCAGCAATAGAATAG			
*astA*	astAF	TGCCATCAACACAGTATATCCG	102	55	(Antikainen et al., 2009)
	astAR	ACGGCTTTGTAGTCCTTCCAT			
*ibeA*	ibeAF	AGGCAGGTGTGCGCCGCGTAC	170	67	(Wang, 2012)
	ibeAR	TGGTGCTCCGGCAAACCATGC			
*iucD*	iucDF	ACAAAAAGTTCTATCGCTTCC	711	51	(Wang, 2012)
	iucDR	CCTGATCCAGATGATGCTC			
*kpsMII*	kpsMIIF	GCGCATTTGCTGATACTGTTG	272	56	(Wang, 2012)
	kpsMIIR	CATCCAGACGATAAGCATGAGCA			
*pap*	papF	GTGGCAGTATGAGTAATGACCGTTA	296	55	(Wang, 2012)
	papR	ATATCCTTTCTGCAGGGATGCAATA			
*cnfI*	cnfIF	AAGATGGAGTTTCCTATGCAGGAG	498	56	(Wang, 2012)
	cnfIR	CATTCAGAGTCCTGCCCTCATTATT			
*sfaS*	sfaSF	GTGGATACGACGATTACTGTG	240	56	(Wang, 2012)
	sfaSR	CCGCCAGCATTCCCTGTATTC			
*malX*	malXF	GGACATCCTGTTACAGCGCGCA	922	63	(Wang, 2012)
	malXR	TCGCCACCAATCACAGCCGAAC			
*ompA*	ompAF	AGCTATCGCGATTGCAGTG	919	56	(Wang, 2012)
	ompAR	GGTGTTGCCAGTAACCGG			
*ompC*	ompCF	GCAGGCCCTTTGTTCGATA	1236	55	(Wang, 2012)
	ompCR	GCCGACTGATTAATGAGGGTTA			
*ompF*	ompF F	GAACTGGTAAACGATACCCACAG	1071	57	(Wang, 2012)
	ompFR	ATTCTGGCAGTGATCGTCCCT			
*K88*	K88F	GCTGCATCTGCTGCATCTGGTATG	792	61	(Yue, 2017)
	K88R	CCACTGAGTGCTGGTAGTTACAGCC			
*K99*	K99F	TATTATCTTAGGTGGTATGG	314	56	(Yue, 2017)
	K99R	GGTATCCTTTAGCAGCAGTATTTC			
*eaeA*	eaeAF	GACCCGGCACAAGCATAAGC	384	59	(Yue, 2017)
	eaeAR	CCACCTGCAGCAACAAGAGG			
*irp2*	irp2F	AAGGATTCGCTGTTACCGGA	267	52	(Wang, 2012)
	irp2R	TCGGCCAGGATGATTCGTCG			

### 2.4 Detection of antibiotic resistance genes and antimicrobial susceptibility testing

In accordance with previously reported methods (Hansen et al., 2004; Park et al., 2006), the following antibiotic resistance‐coding genes were detected by PCR amplification (Table 3): aminoglycoside resistance genes: *aacC2*, *aph(3′)‐la*, *aadA1*, and *aadB*; β‐lactam resistance genes: *bla*_TEM_, *bla*_SHV_, and *bla*_CTX‐M_; tetracycline resistance genes: *tet*(A), *tet*(B), and *tet*(C); quinolone resistance genes: *qnrA*, *oqxAB*, *aac(6′)‐Ib*; and sulphonamide resistance genes: *sul1*, *sul2*, and *sul3*.

**TABLE 3:** Primers targeting *E. coli* antibiotic resistance genes.

Gene	Primer	Sequence (5′→3′)	Length (bp)	Annealing temperature (°C)	Reference
*bla*_TEM_	bla_TEM_ F	CAGAAACGCTGGTGAAAGTA	719	55	(Weng, 2016)
	bla_TEM_ R	ACTCCCCGTCGTGTAGATAA			
*bla*_SHV_	bla_SHV_ F	ATGCGTATATTCGCCTGTG	502	55	(Weng, 2016)
	bla_SHV_ R	CCTCATTCAGTTCCGTTTCC			
*bla*_CTX‐M_	bla_CTX‐M_ F	AGTGAAAGCGAACCGAATC	365	55	(Weng, 2016)
	bla_CTX‐M_ R	CTGTCACCAATGCTTTACC			
*sul1*	sul1F	CATTGCCTGGTTGCTTCAT	238	54	(Weng, 2016)
	sul1R	ATCCGACTCGCAGCATTT			
*sul2*	sul2 F	CATCATTTTCGGCATCGTC	793	54	(Weng, 2016)
	sul2 R	TCTTGCGGTTTCTTTCAGC			
*sul3*	sul3F	AGATGTGATTGATTTGGGAGC	443	54	(Weng, 2016)
	sul3R	TAGTTGTTTCTGGATTAGAGCCT			
*aac(6′)‐Ib*	aac(6*′*)‐IbF	TTGCGATGCTCTATGAGTGGCTA	482	55	(Park et al., 2006)
	aac(6*′*)‐IbR	CTCGAATGCCTGGCGTGTTT			
*oqxAB*	oqxAB F	GATCAGTCAGTGGGATAGTTT	671	50	(Weng, 2016)
	oqxAB R	TACTCGGCGTTAACTGATTA			
*qnrA*	qnrAF	TCAGCAAGAGGATTTCTCA	627	54	(Park et al., 2006)
	qnrAR	GGCAGCACTATTACTCCCA			
*aadA1*	aadA1F	TTTGCTGGTTACGGTGAC	499	56	(Zhang, 2009)
	aadA1R	GCTCCATTGCCCAGTCG			
*aadB*	aadB F	GAGGAGTTGGACTATGGATT	208	53	(Zhang, 2009)
	aadBR	CTTCATCGGCATAGTAAAA			
*aac C2*	aac C2F	GCAATAACGGAGGCAATTCGA	697	56	(Zhang, 2009)
	aac C2R	CTCGATGGCGACCGAGCTTCA			
*aph(3')‐Ia*	aph(3')‐IaF	ATGGGCTCGCGATAATGTC	600	56	(Zhang, 2009)
	aph(3')‐IaR	CTCACCGAGGCAGTTCCAT			
*tet(A)*	tet(A)F	GCTACATCCTGCTTGCCTTC	210	59.5	(Li et al., 2017)
	tet(A)R	CATAGATCGCCGTGAAGAGG			
*tet(B)*	tet(B)F	TTGGTTAGGGGCAAGTTTTG	659	59.5	(Li et al., 2017)
	tet(B)R	GTAATGGGCCAATAACACCG			
*tet(C)*	tet(C)F	CTTGAGAGCCTTCAACCCAG	418	59.5	(Li et al., 2017)
	tet(C)R	ATGGTCGTCATCTACCTGCC			

Antimicrobial susceptibility testing was performed using the Kirby–Bauer test method recommended by the WHO. The results were analyzed and interpreted according to Clinical and Laboratory Standards Institute (CLSI) standard using the disk‐diffusion technique (CLSI, 2018). Paper discs impregnated with 12 antimicrobial agents, namely cefazolin (KZ, 30 μg), ceftazidime (CAZ, 30 μg), cefoperazone (SCF, 75 μg), imipenem(IMP, 10 μg), meropenem(MEM, 10 μg), streptomycin (STR, 10 μg), gentamicin (GEN, 10 μg), doxycycline (DOX, 30 μg), amikacin (AK, 30 μg), ciprofloxacin (CIP, 5 μg), levofloxacin (LEV, 5 μg), and sulfamethoxazole (SMZ, 1.25 μg), were used to determine the antibiotic susceptibilities of 116 *E. coli* isolates. *Escherichia coli* ATCC 25922 was used as the reference strain. A strain was considered multidrug resistant (MDR) when it demonstrated resistance to three or more antimicrobial classes (Sweeney et al., 2018).

### 2.5 Gene sequencing

Selected PCR amplification products were sent to Rui Biotech Co. Ltd. (Beijing, China) for sequencing. The nucleotide sequences obtained were analyzed using the basic local alignment search tool, BLAST‐n (http://www.ncbi.nlm.nih.gov/BLAST) (Liu et al., 2017).

### 2.6 Statistical methods

We used the SPSS software for Windows, version 20.0 (SPSS Inc., Chicago, IL, USA) for statistical analysis (Islam et al., 2017). Comparisons among frequencies of occurrence of each phenotypic or genotypic feature in *E. coli* isolates from samples were carried out by contingency table *χ* ^2^ tests (at *p* < 0.05). The result was considered to be significant at *p* ≤ 0.05.

## 3 RESULTS

### 3.1 Isolation and identification of *E. coli* strains

A total of 116 bacterial strains were isolated from tissues of diseased cattle and sheep in Xinjiang, China (cattle: 94 strains; sheep: 22 strains). They were obtained from diseased tissues such as liver, spleen, lung, heart, brain, and lymph nodes. Among them, 17 strains were isolated in 2015, 20 strains were isolated in 2016, 48 strains were isolated in 2017, 19 strains were isolated in 2018, and 12 strains were isolated in 2019. The sample collection covers the northern and southern Xinjiang regions (including Shihezi, Tacheng, Altay, Ili, Urumqi, Shawan, and Tumshuke). The 16S rRNA gene sequence similarity of the strain to be tested is >97%, which may be related to *E. coli* at the species level. Each isolate was identified by 47 biochemical tests, and the analysis results showed that the biochemical reaction characteristics of the isolate and *E. coli* were basically the same, with a reliability of 98.3%–99.9%.

### 3.2 Detection of virulence determinants by PCR and phylogenetic group determination

The 116 pathogenic *E. coli* isolates were tested for 25 virulence genes, and 17 different virulence genes were detected. The detection rates for *crl*, *hlyE*, *fimH*, *ompA*, *ompF*, *ompC*, and *pap* were relatively high at 97.4%, 94.82%, 92.24%, 75%, 75%, 61.21%, and 52.59%, respectively, whereas the detection rates of other virulence genes were lower (0.86%–47.41%). Virulence genes *agg*, *stx1*, *stx2*, *cnf1*, *sfaS*, *hlyA*, *K88*, and *LT* were not detected. The average carrying rate of virulence genes of bovine‐derived strains was 7.98%, and that of sheep‐derived strains was 3.77%. The two strains were quite different. Among them, bovine‐derived strains could carry up to 12 kinds of virulence genes. *K99*, *ibeA*, *malX*, *irp*2, *kpsMII*, and *eaeA* are unique to bovine‐derived strains, and sheep‐derived strains do not carry. There were differences in the virulence genes carried by isolates in groups A, B1, C, and F, with the number of virulence genes ranging from 2 to 11. Isolates belonging to groups D and E carried four to eight virulence genes, whereas both isolates in group B2 carried 11 virulence genes. Therefore, isolates in group B2 had relatively large numbers of virulence genes compared to those in other phylogenetic groups (Table 4).

**TABLE 4:** Frequency of different virulence genes among the phylogenetic groups of *E. coli* isolated from cattle and sheep.

	Phylogenetic groups								
Virulence gene	Function	No. of isolates (%)	A (*n* = 54)	B1 (*n* = 38)	B2 (*n* = 2)	C (*n* = 8)	D (*n* = 3)	E (*n* = 3)	F (*n* = 8)
*crl*	Adhesin	113 (97.4)	54	38	2	6	3	3	7
*fimH*	Adhesin	107 (92.24)	48	35	2	8	3	3	8
*hlyE*	Toxin	110 (94.82)	51	37	0	8	3	3	7
*astA*	Adhesin	5 (4.3)	0	4	0	0	0	0	1
*ibeA*	Adhesin	5 (4.3)	1	0	2	0	0	0	2
*iucD*	Iron acquisition	50 (43.1)	17	19	2	7	2	0	3
*kpsMII*	Adhesin	5 (4.3)	0	0	2	0	0	0	3
*pap*	Adhesin	61 (52.59)	33	14	2	6	1	1	3
*malX*	Toxin	7 (6.03)	0	0	2	0	0	0	5
*ompA*	Protectin	87 (75)	45	22	2	7	1	3	7
*ompC*	Protectin	71 (61.21)	34	16	2	7	1	3	7
*ompF*	Protectin	87 (75)	45	22	2	7	1	3	7
*K99*	Adhesin	13 (11.21)	11	2	0	0	0	0	0
*irp2*	Iron acquisition	55 (47.41)	35	9	2	6	0	0	3
*ireA*	Iron acquisition	9 (7.76)	0	3	0	4	1	0	1
*fliC*	Adhesin	28 (24.14)	19	5	0	2	0	2	0
*eaeA*	Adhesin	1 (0.86)	0	1	0	0	0	0	0

### 3.3 Detection of antibiotic resistance genes and antimicrobial susceptibility testing

Among the various aminoglycoside resistance genes detected, *aph(3′)‐Ia* was the most common at 63.8% (74/116) and *aadB* was the least common at 6.0% (7/116). Among the various β‐lactam resistance genes, *bla*_TEM_ had the highest detection rate of 73.3% (85/116), and *bla*_SHV_ had the lowest detection rate of 11.2% (13/116). Among the various tetracycline resistance genes, *tet*(A) was detected in 75.9% (88/116) and *tet*(*C*) in 34.5% (40/116) of isolates. Among the various quinolone resistance genes, *aac(6’)‐Ib* showed the highest detection rate of 44.8% (52/116), and *qnrA* showed the lowest detection rate of 19.8% (23/116). Finally, among the various sulfonamide resistance genes detected, *sul2* was most common at 74.1% (86/116) and *sul3* was the least common at 20.7% (24/116) (Table 5).

**TABLE 5:** Distribution of resistance genes in *E. coli* isolates with different antibiotic resistance phenotypes.

Antibiotic category	Resistance genes	No. of isolates with resistance genes	Resistance gene carrier rate (%)
Aminoglycosides	*aadA*	26	22.4
	*aadB*	7	6.0
	*aacC2*	26	22.4
	*aph(3′)‐Ia*	74	63.8
β‐Lactams	*bla*_TEM_	85	73.3
	*bla*_SHV_	13	11.2
	*bla*_CTX‐M_	25	21.6
Tetracyclines	*Tet*(A)	88	75.9
	*Tet*(B)	41	35.3
	*Tet*(C)	40	34.5
Quinolone	*oqxAB*	37	31.9
	*qnrA*	23	19.8
	*aac(6′)‐Ib*	52	44.8
Sulfonamides	*sul1*	32	27.6
	*sul2*	86	74.1
	*sul3*	24	20.7

The average carrying rate of drug resistance genes in bovine‐derived strains was 6.83%, and the average carrying rate of drug‐resistant genes in sheep‐derived strains was 5.5%. The two strains seem to have little difference, the average carrying rate of drug resistance genes in bovine‐derived strains was 6.83%, and the average carrying rate of drug‐resistant genes in sheep‐derived strains was 5.5%. However there is a large difference in the carrying situation between bovine‐derived strains, and the maximum carrying rate of 15 drug resistance genes, and a minimum of 1. Among them, *tet*(C), *qnrA* and *aac(6')‐Ib*, are unique to bovine‐derived strains, but not detected in sheep‐derived strains (Table 6).

**TABLE 6:** Phylogenic, virulence, and resistance characteristics of the 116 *E. coli* isolates.

Phylogenetic group	No. of isolates (%)	Mean virulence score^a^ (±SD)	Resistant isolates^b^ *N* (%)	Mean antibiotic resistance gene score^c^ (±SD)
Cattle strains	94 (81.03)	7.98 (±1.72)	94 (100)	6.83 (±2.96)
Sheep strains	22 (18.97)	3.77 (±1.38)	22 (100)	5.5 (±1.99)
A	54 (46.55)	6.72 (±2.35)	54 (100)	7.5 (±2.36)
B1	138 (32.76)	5.58 (±2.36)	38 (100)	6.39 (±3.06)
B2	2 (1.72)	11 (±0)	2 (100)	4 (±2)
C	8 (6.9)	8.38 (±2.06)	8 (100)	6.25 (±2.59)
D	3 (2.59)	5.33 (±1.25)	3 (100)	5.33 (±3.09)
E	3 (2.59)	7.3 (±0.94)	3 (100)	1.33 (±0.47)
F	8 (6.9)	8.13 (±2.09)	8 (100)	7.75 (±1.48)

^a^ Mean virulence score: the mean number of virulence genes carried by isolates in the group.

^b^ Resistant isolates: the number of isolates with resistance to at least three antimicrobial agents.

^c^ Mean antibiotic resistance gene score: the mean number of antibiotic resistance genes carried by isolates in the group.

**FIGURE 1:**
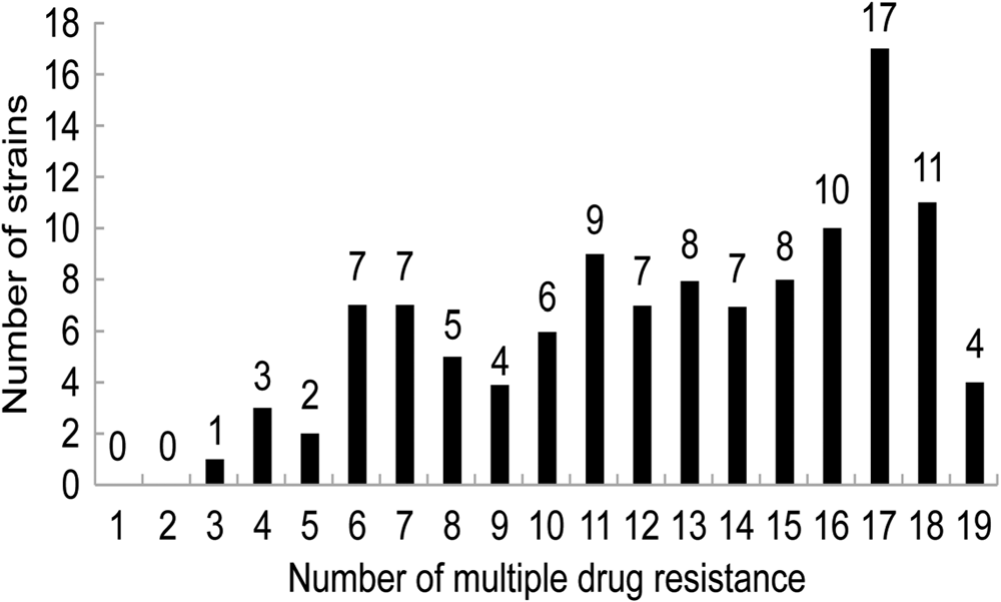
Results of multiple antibiotic resistance analysis of pathogenic *E. coli*.

All of the 116 *E. coli* isolates exhibited MDR (defined as resistance towards three or more antibiotics) (Figure 1). In particular, 41.4% of strains were resistant to more than 10 antibiotics. The rates of resistance of the isolates to antibiotics were as follows: streptomycin: 81.9%; gentamicin: 75%; sulfamethoxazole: 68.1%; cefazolin: 67.2%; ciprofloxacin: 56%; levofloxacin: 52.6%; doxycycline: 51.7%; cefoperazone, ceftazidime, and amikacin: 46.6%–31.9%; imipenem:5.17%, and meropenem:3.4%.

## 4 DISCUSSION

*Escherichia coli* infections are among the three major bacterial infections that are detrimental to livestock breeding around the world. *Escherichia coli* can be either symbiotic or pathogenic, and the phylogenetic positions of these bacteria are determined by different gene cluster combinations that are critical for understanding the pathogenesis of *E. coli* and host–*E. coli* interactions (Irino et al., 2007). Research on *E. coli* genomes has indicated that phylogenetic groupings of strains may be related to isolation sources (Clermont et al., 2013). Previous studies have shown that pathogenic *E. coli* from cattle, non‐cattle (Karczmarczyk et al., 2011), and birds (Cunha et al., 2014) mainly belong to groups A and B1. In this study, the majority of the 116 pathogenic *E. coli* strains isolated from diseased cattle and sheep in Xinjiang also belonged to groups A and B1, and the minority belonged to groups B2, C, D, E, and F.

*Escherichia coli* is a pathogen of worldwide significance, resulting in diseases such as dysentery (e.g., shigellosis), neonatal meningitis (associated with the presence of a K1 capsular polysaccharide), and haemolytic‐uremic syndrome (associated with *E. coli* O157:H7). Virulence is generally related to the presence of specific gene clusters termed “pathogenicity islands” (Groisman & Ochman, 1996; Hacker & Kaper, 2000). Cattle serve as typical reservoirs for intestinal pathogenic *E. coli* and extraintestinal pathogenic *E. coli* virulence factors. The results of this study indicate that the distribution of virulence factors in the two host species of bovine and sheep *E. coli* showed different patterns. For instance, the six most common virulence genes (*K99*, *ibeA*, *malX*, *irp2*, *kpsMII*, and *eaeA*) in cattle were not detected in isolates from sheep. In particular, *irp2*, which encodes iron‐regulatory protein 2, was usually present in the isolates from cattle, but it was not detected in isolates from sheep (*p* < 0.05). Despite no clear influence on the iron‐uptake ability of *E. coli*, the presence of siderophores and haemolysins confers additional virulence mechanisms to extra‐intestinal infections by the pathogen, such as mammary infections (Kempf et al., 2016). Specifically, curli‐encoding *crl* had the highest detection rate (97.4%). Nesta et al. (2014) asserted that the pathogenicity of *E. coli* strains is determined, to a certain extent, by virulence factors encoded in the *E. coli* genome. In *E. coli*, curli may be involved in adherence of septicemic *E. coli* (SePEC) to the mucosal extracellular matrix after damage of the epithelium, as well as the formation of biofilms on organic (animals or plants) and non‐organic surfaces. The ability to form biofilms in epithelial cells is related to their persistence, leading to the severity and recurrence of the disease. Curly pili is one of the most important components of biofilm extracellular matrix (Qasemi et al., 2021). Our results indicate that curli is a virulence gene widely present in pathogenic *E. coli* from cattle and sheep in Xinjiang. This may be one of the important virulence factors produced by the disease. During host invasion, local adherence and colonization by *E. coli* occur first, facilitating invasion of host cells and the generation of toxic metabolites. The interaction among virulence factors is also the main cause of bacterial pathogenicity (Groisman & Ochman, 1996).

*Escherichia coli* is regarded as a reservoir of antibiotic resistance determinants. This bacterium readily develops antibiotic resistance and is capable of transferring antibiotic resistance factors to other pathogenic microbes in gastrointestinal tracts (Blake et al., 2003). Antibiotic resistance rates of *E. coli* have risen steadily over the years. In a study by Massot et al. (2016) on the trends of *E. coli* antibiotic resistance rates in Paris, France, over a 30‐year period, antibiotic resistance was shown to increase from 22.6% in 1980 to 31.8% in 2000 and 44.8% in 2010. Another study on *E. coli* isolated from cattle between 2009 and 2016 in Sichuan, China, showed that antibiotic resistance of *E. coli* towards 27 drugs gradually increased from 2009 to 2016 (Chen et al., 2017). The isolates exhibited rates of resistance of 81.9%–68.1% to streptomycin, gentamicin, and sulfamethoxazole and showed a range of resistance rates for the other tested antibiotics. The results (resistant, intermediate, and sensitive) were interpreted by following the guidelines of the CLSI (CLSI, 2018), and where not possible, according to the European Committee on Antimicrobial Susceptibility Testing (EUCAST, 2019). Isolates showing resistance to three or more classes of antibiotics were recorded as MDR (Sweeney et al., 2018). Resistance to β‐lactams was highest, but the most commonly detected antibiotic resistance gene was tetracyclines resistance‐encoding *tet*(A), which indicates a difference between antibiotic resistance phenotypes and the presence of antibiotic resistance genes. Although 88% of the isolates carried *tet*(A), only 60% of the isolates exhibited resistance to tetracyclines, which might be due to differences in the expression of *tet*(A) among the isolates. These results indicate a high degree of antibiotic resistance in the *E. coli* isolates obtained from cattle and sheep in Xinjiang.

In a previous study, all isolates obtained in this study exhibited MDR, and isolates with a higher degree of MDR carried a larger number of virulence genes. For instance, isolate N‐3 carried 10 virulence factors and 10 antibiotic resistance genes. A more detailed analysis revealed an association between β‐lactamase genes and virulence genes, with tetracyclines and sulphonamides showing associations with virulence genes in this study. The relationship between antibiotic resistance factors and virulence genes may depend on the origin of the strain, type of antibiotic, type of virulence gene, and even geographical location of isolate source (Koczura et al., 2013). As shown in Table 6, the number of virulence genes carried by isolates in groups C and F are similar, and isolates in group F carried a larger number of antibiotic resistance genes. Several combinations of virulence and antibiotic resistance genes were observed and lacked significant patterns, partly limited by the number of strains analyzed. In addition, the number of B2 and D groups in the strains was small and could not be statistically analyzed. The emergence of relationships between antibiotic resistance and virulence genes suggests that resistant isolates are more virulent than susceptible isolates. Because of the resistance, bacteria can persist longer in the host than susceptible bacteria and may therefore be difficult to treat. According to these results, cattle isolates are more virulent than sheep isolates, possibly due to excessive use of anti‐microbials without proper diagnosis. These strains were found to be more toxic and resistant to multiple drugs, leading to initiation of several tests related to the development of animal husbandry in Xinjiang.

## 5 CONCLUSION

This study revealed that the prevalence of multi‐antibiotic‐resistance, and virulence gene carrier rate were generally high among *E. coli* isolates from cattle and sheep suspected of *E. coli* disease in Xinjiang and is increasing year by year. *crl* has a high carrying rate in this study, and it plays an important role in the colonization and initiation of the infection. The virulence gene carrying rate of cattle strains is much higher than that of sheep strains. Regular screening and monitoring of antibiotic resistance and virulence genes related to *E. coli* isolates are essential for the implementation of intervention programmes aiming to reduce the risk of coliform disease. In Xinjiang and other parts of China, a holistic approach is needed to prevent and control cattle and sheep *E. coli* disease.

## AUTHOR CONTRIBUTIONS

Xiaoxiao Gu, Zhou X, Yingjin Chai, and Han ML carried out the molecular genetic studies, participated in the primers sequence alignment, and drafted the manuscript. Xue Ma, Qin Wu, Yiheng Cao, Liyuan Zhang, and Qiaoxiaoci Tao carried out the sampling and culture method. Jie Li, Xin Huang, Tongzhong Wu, Fagang Zhong, and Xingxing Zhang participated in the design of the study, performed the statistical analysis, and wrote the manuscript. All authors read and approved the final manuscript.

## CONFLICT OF INTEREST STATEMENT

The authors declare no conflict of interest.

### ETHICS STATEMENT

All animal experiments were reviewed and approved by the ethics committee of the First Affiliated Hospital of Medical College, Shihezi University, China (approval number: A2019‐149‐01).

### PEER REVIEW

The peer review history for this article is available at https://publons.com/publon/10.1002/vms3.1101.

## Data Availability

Data sharing not applicable as no new data were generated, and the article describes entirely theoretical research.
